# Construction and validation of an immunity-related prognostic signature for breast cancer

**DOI:** 10.18632/aging.103952

**Published:** 2020-11-07

**Authors:** Tao Zhu, Juyan Zheng, Shuo Hu, Wei Zhang, Honghao Zhou, Xi Li, Zhao-Qian Liu

**Affiliations:** 1 Department of Clinical Pharmacology, Hunan Key Laboratory of Pharmacogenetics, and National Clinical Research Center for Geriatric Disorders, Xiangya Hospital, Central South University, Changsha 410008, P.R. China; 2 Institute of Clinical Pharmacology, Engineering Research Center for Applied Technology of Pharmacogenomics of Ministry of Education, Central South University, Changsha 410078, P.R. China; 3 Department of Nuclear Medicine, Key Laboratory of Biological Nanotechnology of National Health Commission, Xiangya Hospital, Central South University, Changsha 410008, P.R. China

**Keywords:** tumor immunology, breast cancer, prognostic signature, immunity-related gene, transcription factor

## Abstract

Breast cancer is one of the most lethal malignancies among women, and understanding the effects of host immunity on disease progression offers the potential to improve immunotherapies against it. Here, we constructed an immunity-related gene (IRG)-based prognostic signature to stratify breast cancer patients and predict their survival. We identified differentially-expressed genes by analyzing the breast cancer transcriptome data from The Cancer Genome Atlas. Univariate Cox regression revealed 179 survival-correlated IRGs, 12 of which we used to construct an immunity-based prognostic signature that stratified breast cancer patients into high- and low-risk groups. The signature was an independent predictor for survival and was validated in an independent dataset. We also investigated the correlations between our prognostic signature and immune infiltrates and found that signature-derived risk scores correlated negatively with infiltration of B cells, CD4+ T cells, CD8+ T cells, neutrophils and dendritic cells. Our results show that the proposed prognostic signature reflects the tumor immune microenvironment, which makes it a potential indicator for survival that warrants further research to assess its clinical utility.

## INTRODUCTION

Breast cancer is the most frequently diagnosed malignancy and one of the leading causes of cancer-related mortality among women. It is estimated that in 2020 breast cancer will account for ~30% of all new female cancer diagnoses, and for ~15% of all female cancer deaths [1]. Patient survival is still threatened by resistance to treatment, relapse and metastasis, despite advances in multidimensional treatment including surgery, chemotherapy and targeted therapy. Breast cancer is a heterogeneous disease that can be differently subtyped based on disparate classifications [2–4]. Identification of specific biomarkers of breast cancer can help to predict and monitor disease progression, and to reduce aggressive cases through early intervention.

The interplay between tumors and host immunity plays a critical role in breast cancer biology [5]. Immunoediting has emerged as a relevant hallmark of cancer, which enables tumor cells to escape from immune attack by concealing their surface antigens or switching off functions of immune cell effectors [6, 7]. Cancer immunoediting can be described by three phases termed elimination, equilibrium, and escape [8]. In the elimination phase, tumor cells are recognized and killed by innate immune cells. If tumor cells evolve and reduce their immunogenicity, resistance will be acquired as a result, which means they can survive host immune responses and equilibrium is reached. During the escape phase, nascent cancer cells can't be eradicated by immune cells, giving rise to rapid progression [8]. Hence, breast cancer progression is subject to the activity of the immune system, which varies among individuals. A recent study showed that based on immune activity, basal-like triple-negative breast cancer (TNBC) can be subclassified into immunosuppressed and immune-activated subtypes with distinct prognoses [3], suggesting a potential immunological gene-based signature in breast cancer that may be exploited for prognostic benefit.

Recent studies have improved our understanding of the mechanisms underlying carcinogenesis and have propelled the development of anti-cancer drugs, such as cancer vaccines [9–11]. Genome-wide profiling has helped to gain insights into disease progression and is an efficient way to deepen our understanding of cancer biology [12–16]. In this study, our aim was to identify high-risk breast cancer patients based on a prognostic signature of immunity-related genes (IRGs), which we constructed by integrating transcriptomic data with clinical information to screen for survival-associated IRGs. We also investigated potential regulatory mechanisms by analyzing associated transcription factors and validated the reliability of the IRGs-based prognostic signature in an independent cohort. Lastly, to investigate if the signature could reflect the immune microenvironment of breast cancer, we analyzed the correlations between risk scores derived from the IRGs-based signature and immune cell infiltration.

## RESULTS

### Identification and functional annotation of differentially-expressed IRGs

**Figure 1:**
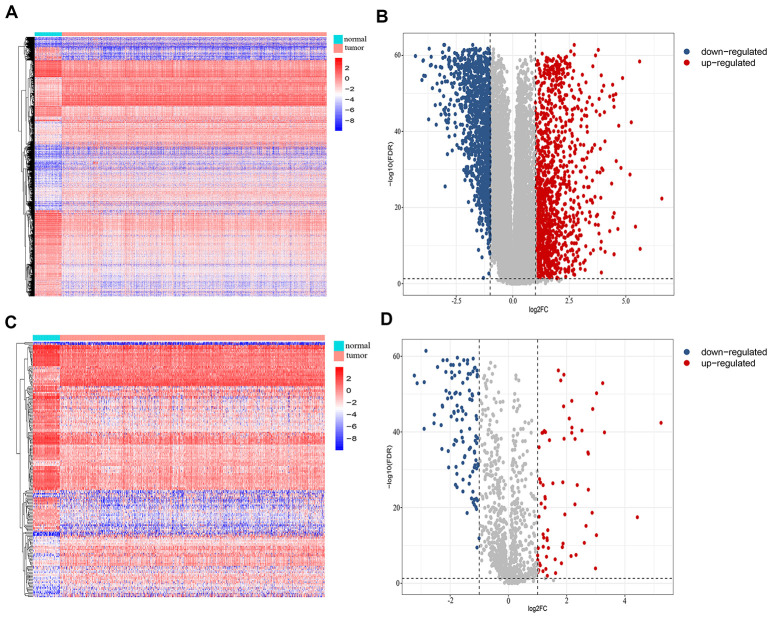
**Identification of differentially-expressed immunity-related genes in breast cancer.** (**A**) Heatmap and (**B**) volcano plot showing differentially-expressed genes between breast and non-malignant tissues. (**C**) Heatmap and (**D**) volcano plot of differentially-expressed immunity-related genes in breast cancer.

**Figure 2:**
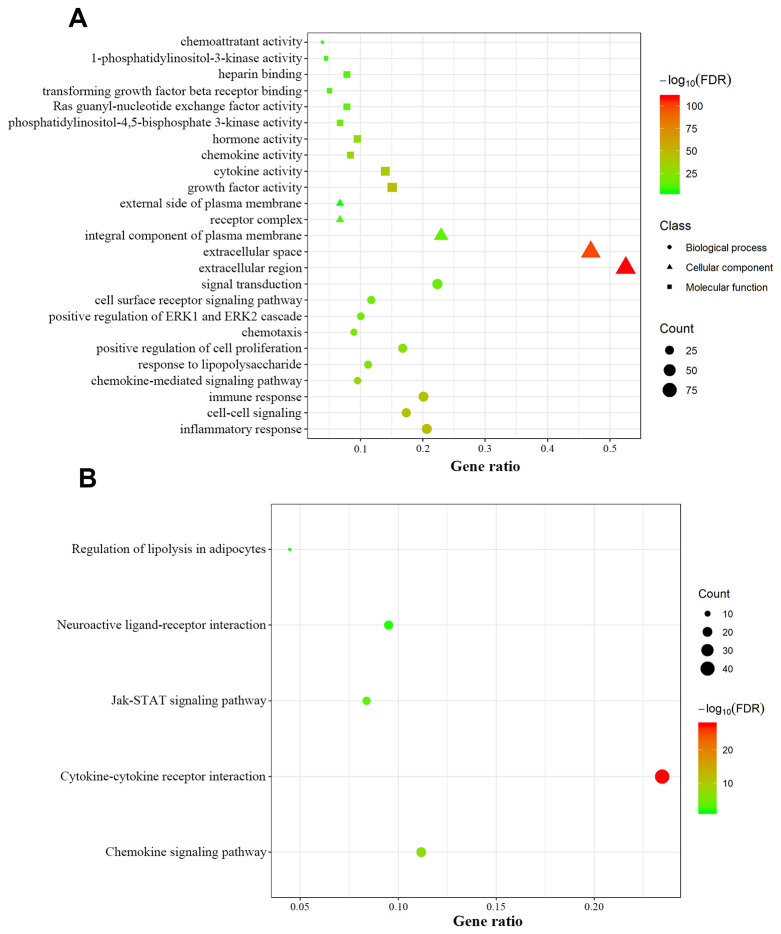
**Functional annotation of differentially expressed immunity-related genes.** (**A**) Enriched Gene Ontology terms including biological process (dots), cellular component (triangles) and molecular function (squares). (**B**) Enriched KEGG pathways.

We retrospectively analyzed the TCGA breast cancer dataset, which included 1104 tumor samples and 113 normal adjacent samples, to identify differentially-expressed genes. A total of 2459 genes were identified as differentially expressed, with 1231 being upregulated and 1228 downregulated (Figure 1A, 1B). Since host immunity contributes to determining neoplasm destiny, here we focused on immunity-related genes. From these differentially-expressed genes, we extracted IRGs and obtained 69 upregulated and 110 downregulated IRGs (Figure 1C, 1D), which are listed in Supplementary Table 1. To gain insight into the biological roles of the 179 differentially-expressed IRGs, we performed GO and KEGG pathway enrichment analysis, which identified 64 relevant GO terms and five relevant KEGG pathways (Supplementary Table 2). As expected, GO analysis indicated that major terms enriched in the biological process category were signal transduction, inflammatory response and immune response. The most enriched cellular component terms were “extracellular region” and “extracellular space”. For the molecular function category, the primary enriched terms were growth factor activity, cytokine activity and chemokine activity (Figure 2A). KEGG pathway analysis revealed that the differentially-expressed IRGs were involved in cytokine-cytokine receptor interactions, chemokine signaling, and neuroactive ligand-receptor interactions (Figure 2B).

### Screening of survival-associated IRGs

**Figure 3:**
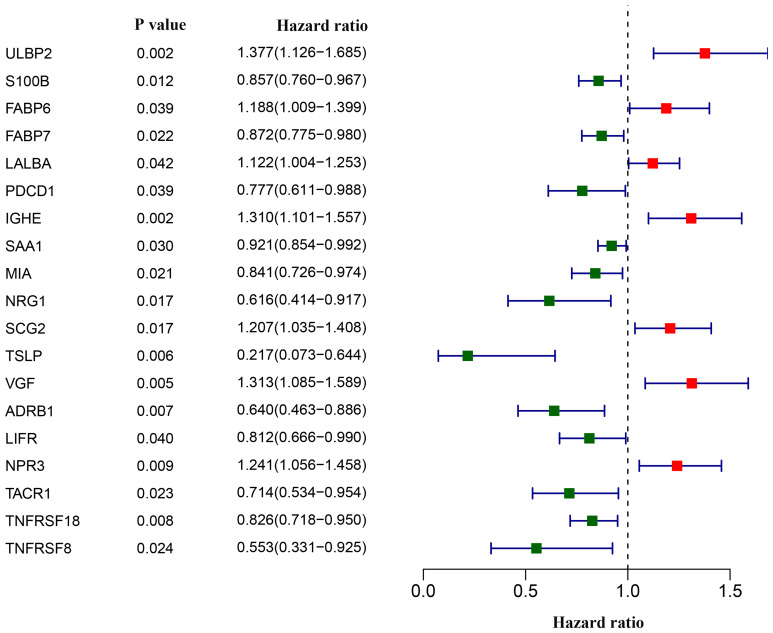
**Screening of differentially expressed immunity-related genes correlated with overall survival in breast cancer.** The forest plot shows hazard ratios of each gene.

We next asked whether and which of the aforementioned 179 differentially-expressed IRGs could aid in disease management in breast cancer patients. Univariate Cox analyses revealed that 19 IRGs were associated with overall survival. Most of the 19 survival-associated IRGs were protective factors, as indicated by their hazard ratios (Figure 3).

### Transcription factors involved in regulation of survival-associated IRGs

**Figure 4:**
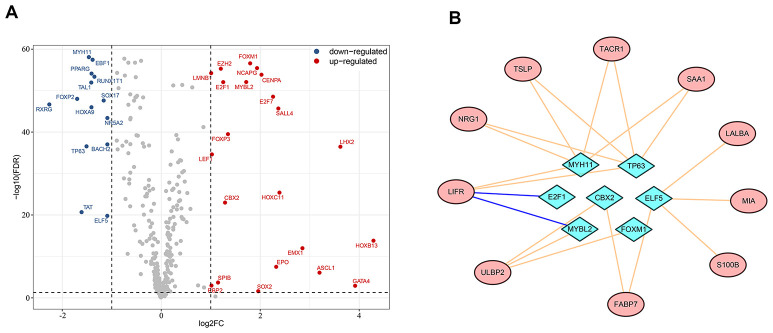
**Regulatory network of survival-associated immunity-related genes based on differentially-expressed transcription factors.** (**A**) Highlight of up- and down-regulated transcription factors in breast cancer. (**B**) The regulatory network between transcription factors and prognostic immunity-related genes. Survival-associated immunity-related genes are shown in ellipses and differentially-expressed transcription factors in diamonds. A pink line represents positive regulation and a blue line represents negative regulation.

Dynamic alterations in the expression level of transcription factors can affect immune cell fates and tumor development and progression [17–19]. To investigate potential regulatory mechanisms of the survival-associated IRGs, we extracted candidate transcription factors from all differentially-expressed genes to analyze the associations in gene expression between transcription factors and survival-associated IRGs. Of the 36 differentially-expressed transcription factors we identified (Figure 4A), E2F1 and MYBL2 were negatively associated with LIFR expression, while CBX2, ELF5, FOXM1, MYBL2, MYH11 and TP63 were in positive associations with at least one of the survival-associated IRGs. Figure 4B shows a modulatory network illustrating the regulation between the above seven transcription factors and survival-associated IRGs.

### Construction of the immunity-related prognostic model

**Figure 5:**
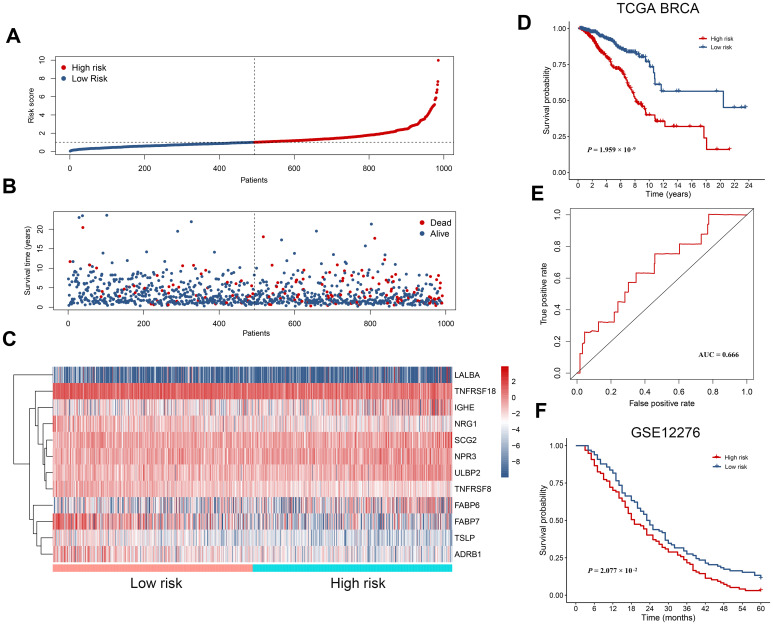
**The prognostic immunity-based signature in breast cancer.** (**A**) Distribution of risk score derived from the signature. Patients are ranked according to the corresponding risk score. (**B**) Survival status of breast cancer patients. They are ranked in the same way as in (**A**). (**C**) Heatmap showing expression of the 12 hub immunity-related genes in different risk groups. (**D**) Kaplan-Meier survival curves for patients in the TCGA BRCA dataset. Patients are assigned into high and low risk groups according to the median risk score. (**E**) The receiver operating characteristic (ROC) curve showing a prognostic value of the immunity-based signature. (**F**) Kaplan-Meier survival curves for patients in the validation dataset (GSE12276).

The 19 survival-associated IRGs were subject to further analysis in order to construct an immunity gene-based prognostic signature. According to results of multivariate Cox regression analysis, 12 hub IRGs were eligible and we used them to construct a prognostic signature, which we in turn used to derive a risk score for each patient. This risk score was the sum of products of the expression value and the regression coefficient of each gene, per the following formula: risk score = (0.2699 * expression level of ULBP2) + (0.1645 * expression level of FABP6) + [(-0.1348) * expression level of FABP7] + (0.1831 * expression level of LALBA) + (0.2911 * expression level of IGHE) + [(-0.3570) * expression level of NRG1] + (0.1907 * expression level of SCG2) + [(-0.8889) * expression level of TSLP] + [(-0.2905) * expression level of ADRB1] + (0.1408 * expression level of NPR3) + [(-0.1646) * expression level of TNFRSF18] + [(-0.6666) * expression level of TNFRSF8]. As expected, we could see in the formula that risky genes with a hazard ratio > 1 had a positive coefficient, while genes with a hazard ratio < 1 had a negative coefficient (Figure 3). With the median risk score as the cutoff value, all breast cancer patients were assigned to either a high-risk or a low-risk group (Figure 5A). Figure 5B, 5C show an overview of the overall survival and hub IRG expression of these groups. Patients in the high risk group had poorer overall survival compared with that of the low risk group (Figure 5D). Receiver operating characteristic (ROC) curve analysis suggested good sensitivity and specificity of the prognostic model (Figure 5E). We next validated the 12 hub IRGs-based prognostic model in an independent breast cancer cohort, for which we calculated patient risk scores to group the patients into low- and high-risk groups using the median risk score as a cutoff. In line with the survival analysis result of the TCGA BRCA cohort, patients with a higher risk score showed poorer overall survival than those with a lower risk score (Figure 5F). These data collectively revealed that the 12 hub IRGs-based prognostic model might be useful in evaluating the risk degree of a breast cancer patient while predicting overall survival time.

### IRGs as an independent risk factor for breast cancer patients

**Figure 6:**
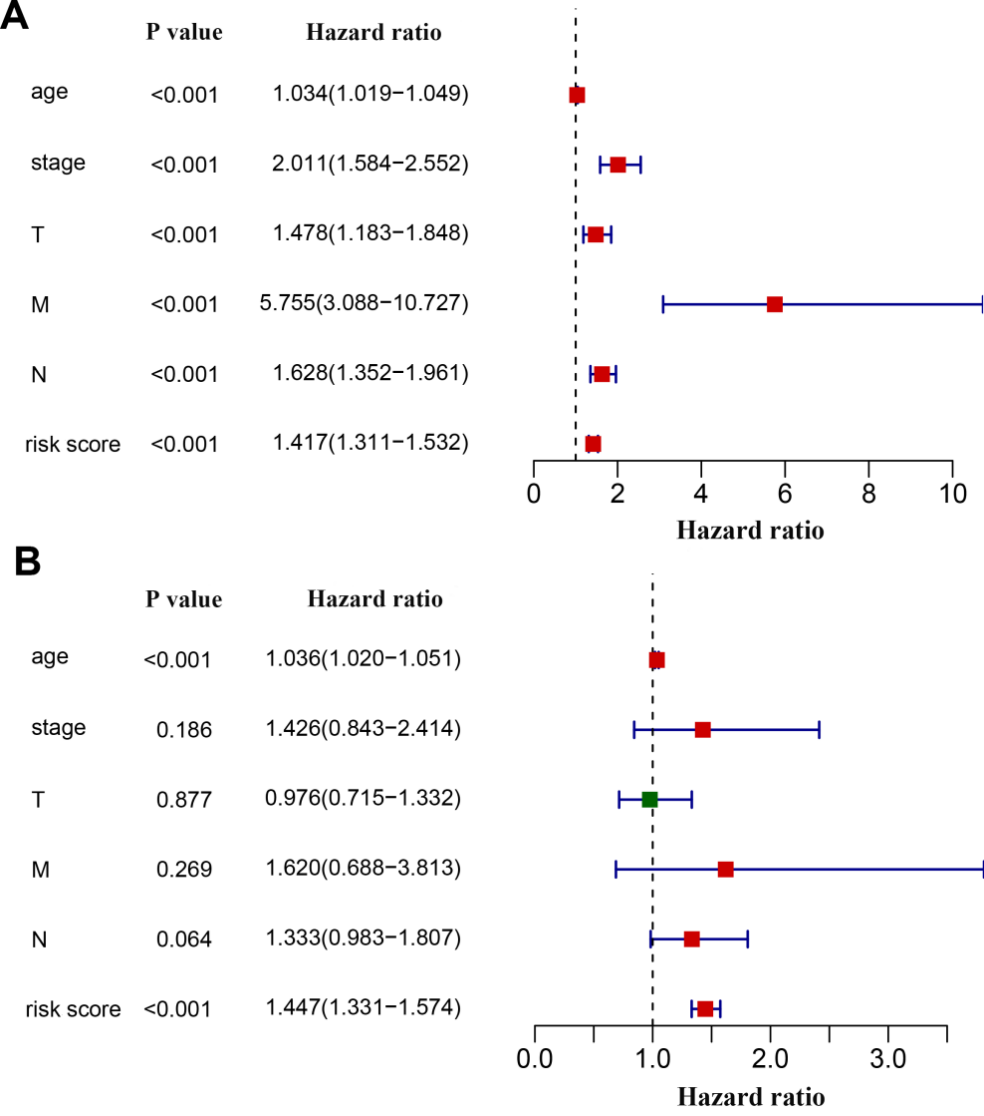
**Effects of critical clinical characteristics on patients’ overall survival in breast cancer.** (**A**) Forest plot showing prognostic values of age, stage, TNM staging and the immunity-based signature-derived risk score. (**B**) Forest plot showing the prognostic value of age, stage, TNM staging and risk score as an independent factor.

We next investigated whether the power of the immunity-related prognostic model was influenced by confounding factors. In addition to the risk score derived from the prognostic model, relevant clinical characteristics including age, tumor stage and TNM classification were used for univariate analysis. As expected, all of these factors were associated with a poor prognosis, with a metastatic disease being the most hazardous factor (Figure 6A). According to multivariate regression, age (HR = 1.036, 95% CI: 1.020−1.051, P < 0.001) and risk score (HR = 1.447, 95% CI:1.331−1.574, P < 0.001) were independent factors correlated with shorter overall survival (Figure 6B). However, the hazard ratios indicated that the prognostic model-derived risk score was a more powerful factor to assess patient prognosis. We also excluded patients without tumor stage information to investigate the correlation of the derived risk score with tumor stage. We compared the risk scores of the Stage i-iia group to those of the Stage iib-iv group and found that risk scores were higher in the latter than in the former (Supplementary Table 3).

### Associations of immunity-related signature with tumor-infiltrating immune cells

**Figure 7:**
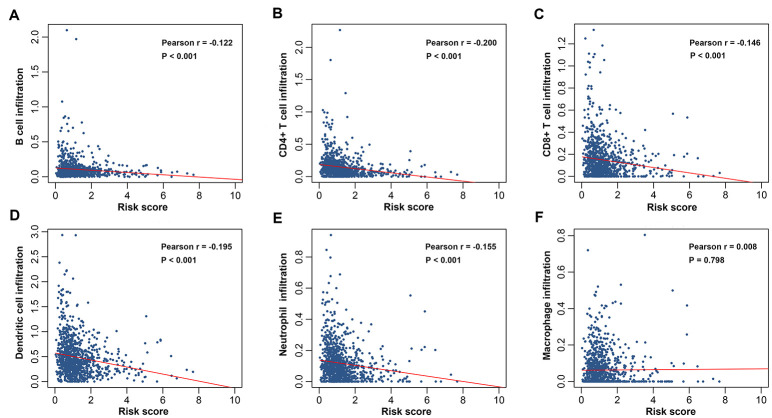
**Correlations between the prognostic signature-derived risk score and infiltration abundances of multiple immune cells.** (**A**) B, (**B**) CD4+ T, (**C**) CD8+ T, and (**D**) dendritic cells; (**E**) neutrophils; (**F**) macrophages.

**Figure 8:**
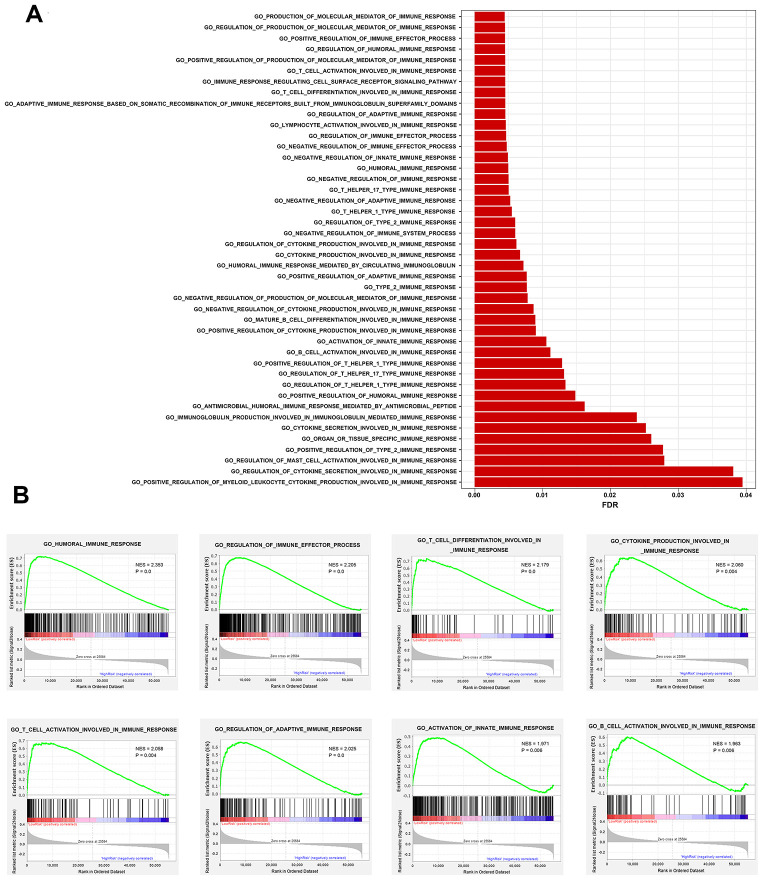
**Comparison of immune traits between high-risk and low-risk groups via gene set enrichment analysis.** (**A**) Enriched immunity-related GO terms. (**B**) Representative immunity-related terms positively correlated with low risk.

To explore whether the immunity-related signature could reflect infiltration degree in the tumor microenvironment of immune cells, we further analyzed the associations between the immune prognostic model-derived risk score and immune infiltrates. As shown in Figure 7A–7E, infiltration of B cells, CD4+ T cells, CD8+ T cells, neutrophils and dendritic cells was negatively correlated with risk score, suggesting that there are fewer tumor infiltrates in high risk patients. These results are consistent with previous findings [20–24]. However, we didn’t find an association between macrophage infiltration and risk score (Figure 7F). We also investigated whether differences in the immune phenotype could be observed between high- and low-risk groups. According to GSEA results, a total of 44 immunity-related GO terms were enriched (Figure 8A), indicating varied intensities of immune response between these two groups. Eight representative immunity-related terms correlated positively with low risk (Figure 8B), which included humoral immune response, regulation of immune effector process, T cell activation and differentiation involved in immune response, cytokine production involved in immune response, regulation of adaptive immune response, activation of innate immune response, and B cell activation involved in immune response. These data suggest that the low risk estimated for many breast cancer patients using our prognostic signature may be attributed to a hyperactivated immune response in the tumor microenvironment.

## DISCUSSION

Breast cancer is a heterogeneous disease that severely threatens the health of females. Although advances in early diagnosis and multiple therapies have contributed to large improvements in survival rate, no specific risk factors have been identified yet for the majority of breast cancer patients [5], and patients afflicted with metastatic disease will eventually succumb to it [25]. The immune system is actively involved in the development and progression of many solid tumors, including those of breast cancer. Accumulating evidence highlights that the response to antitumor therapy and overall survival of breast cancer patients is subject to host immunity [26]. In this regard, an immunity-based prognostic signature can be rationally applicable to identify patients with poor survival in advance. In this study, using two breast cancer cohorts containing over 1000 patients, we constructed and validated a prognostic signature based on the expression of 12 immunity-related genes. According to the risk score yielded from the prognostic signature, patients could be stratified into subgroups with distinct survival outcomes. Moreover, we found that this immune signature was negatively associated with immune infiltrates in the tumor microenvironment. These findings suggest that our immunity-related signature could be used effectively to evaluate prognosis and provide potential immunotherapeutic targets for breast cancer patients.

The current study identified 179 differentially-expressed IRGs between tumoral and normal tissues, which might be involved in breast cancer initiation and progression. Accumulating studies establish that chronic inflammation can trigger tumor progression and an inflammatory surrounding microenvironment is indispensable to all tumors [27–29]. Cytokines, regardless of their source, play opposing roles in cancer progression, either facilitating or suppressing it. A delicate balance between antitumor immunity and tumor-promoting inflammation is achieved through cytokines and chemokines released by immune cells, tumor cells and other components in the tumor microenvironment [27, 30]. Our functional enrichment analysis revealed that the differentially-expressed IRGs participated in extracellular region components, inflammatory response, immune response, cytokine activity, interaction of cytokines with their receptors, and chemokine signaling pathways, which reflected the intricate relationship between inflammation, immunity and cancer, and the involvement of the differentially-expressed IRGs in breast cancer initiation and progression. Increasing evidence demonstrates a critical role of signal transducer and activator of transcription (STAT) proteins in determining whether immune responses in the surrounding milieu are pro-carcinogenic or anti-carcinogenic [31, 32]. In particular, cytokine-activated STAT3 can promote cancer proliferation and invasion while suppressing anti-tumor immunity by regulating the expression of immune checkpoint proteins PD-1, PD-L1 and CTLA4 [31, 33–35]. According to the KEGG pathway analysis in our study, enrichment of Jak-STAT signaling suggested that differentially-expressed IRGs disrupt the balance between anti-tumor immunity and tumorigenesis.

In order to answer whether and which of the differentially-expressed IRGs have an effect on the clinical outcomes of breast cancer patients, we assessed the associations between expression levels of these IRGs and patient overall survival. Out of the 179 IRGs, 7 were positively and 12 were negatively associated with favorable prognosis. The TFs-based regulatory network we built for these 19 survival-associated IRGs showed that MYH11, TP63 and ELF5, were critical regulators of the survival-associated IRGs. To date, the function of MYH11 in breast cancer remains elusive. Increasing evidence demonstrates that TP63 proteins are tumor suppressors which block the metastatic potential of tumor cells [36, 37], although an oncogenic TP63 isoform has also been identified [38]. ELF5 was established as a suppressor of epithelial-mesenchymal transition that inhibited metastasis in breast cancer [39]. We found that MYH11, TP63 and ELF5 were downregulated in breast cancer tissues, which is consistent with previous findings highlighting TP63 and ELF5 as tumor suppressors. Moreover, targets of MYH11 and TP63 (LIFR, NRG1, TSLP, TACR1), and of ELF5 (LALBA, MIA, S100B, FABP7) in the network were also downregulated (except LALBA), which were indicators of better overall survival according to univariate Cox regression. Further, positive associations between the hub TFs (MYH11, TP63 and ELF5) and their targets were found and showed in the network. Collectively, our data suggest that altered expression of certain IRGs correlated with survival in breast cancer patients, perhaps owing to changes in expression of tumor-related TFs, and that the tumor suppressors TP63 and ELF5 can exert their anti-tumor effects by disrupting immune responses in the tumor microenvironment. Further evidence is needed to experimentally elucidate underlying regulatory mechanisms of these immunity-related genes.

Our immunity-based prognostic signature consists of 12 survival-associated IRGs. Among them, ULBP2, TSLP, TNFRSF8 and TNFRSF18 are involved in intercellular cytokine-mediated communications. ULBP2 is one of the NKG2D ligands to cause release of multiple cytokines and chemokines that help to activate NK cells. However, soluble ULBP2 secreted by tumor cells also contributes to evasion of immunosurveillance. Recent studies have found that elevated expression of ULBP2 is an indicator of poor prognosis in ovarian and pancreatic cancer [40, 41]. Similarly, our analysis revealed that ULBP2 is upregulated in breast cancer with detrimental effects on patient overall survival. TNFRSF8 is a tumor necrosis factor with unclear contributions to breast cancer. Here, we found a decreased expression of TNFRSF8, similar to a recent immunohistochemistry-based study that reported a lack of CD30 expression in breast cancer [42]. Moreover, its downregulation was found to be associated with poor prognosis. Interestingly, here we found that TSLP expression is reduced in breast cancer patients and correlates positively with a favorable prognosis, both in disagreement [43] and agreement [44] with previous reports. Further efforts are required to better understand TSLP functions in breast cancer. In line with previous research [45], we confirmed here that NRG1 is downregulated in breast cancer tissues, with detrimental effects on long-term survival. FABP7 was previously associated with lower lymph node stage and a longer disease-free survival [46], which supports our finding that high FABP7 expression contributes to a low risk score. Serum alpha-lactalbumin (LALBA) was previously identified as a marker for breast cancer [47]. We found that high LALBA expression was a hazardous prognostic factor. The roles of other IRGs in our signature in breast cancer, including IGHE, SCG2, NPR3 and FABP6, require further clarification.

We also investigated the relationships between risk score derived from IRGs-based signature and immune cell infiltration to figure out if the signature could reflect the immune microenvironment of breast cancer. Our data revealed that patients with a higher risk score had decreased infiltration degrees of B cells, CD4+ T cells, CD8+ T cells, neutrophils, and dendritic cells. The negative associations of risk score with immune cell infiltration suggested that the signature might serve as a predictor for local immune responses in the tumor bed. Tumor infiltrating B cells express and secrete antibodies to facilitate lysis and apoptosis of tumor cells [48]. The direct and indirect cytotoxic effects of CD8^+^ T cells and CD4^+^ T cells support the finding that their infiltration is correlated with favorable survival outcomes [20, 49]. Neutrophils play a critical role in activating and regulating immune cells as well as their effector functions [50]. A recent study has shown that infiltration by myeloperoxidase-positive neutrophils is an independent prognostic factor associated with a better overall survival in breast cancer [51]. Dendritic cells-mediated priming of T cells is a crucial step in antitumor immunity. It was found that a defective function of dendritic cells in patients with early breast cancer might be important for tumor progression [52]. Taken together, high risk score may be ascribed to, at least in part, unbalanced immune infiltrates and dysfunctional immune responses in the milieu surrounding tumors. It is proposed here that strategies attempting to enrich immune cells in the tumor microenvironment may have positive effects in breast cancer immunotherapy.

Although an immunity-related gene signature constructed based on IRGs differentially expressed between high- and low-immune-score groups in breast cancer was previously reported [53], our study differs from it in that the current prognostic signature was computed on the basis of differentially-expressed IRGs between breast cancer tissues and adjacent normal tissues. This is a well-established approach to identify candidate genes involved in tumorigenesis. Some limitations exist in this study. First, although we validated the signature in an independent cohort, a larger sample size would allow for stronger validation. Second, the prognostic signature should be applied in clinical contexts to test its efficacy. Third, since our immunity-based prognostic signature is constructed using a set of specific genes, further experimental studies are warranted to reveal their functions in breast cancer initiation and progression. Fourth, breast cancer is a heterogeneous disease; thus, whether our prognostic signature is applicable to different histological types of breast cancer remains to be further evaluated in subsequent studies with samples from various histopathologic types.

## MATERIALS AND METHODS

### Data acquisition and processing

RNA-sequencing data of TCGA BRCA and relevant clinical information were downloaded from UCSC Xena (https://xena.ucsc.edu/). A validation cohort (GSE12276) was downloaded from Gene Expression Omnibus (GEO). In these two breast cancer datasets, only patients with complete survival information and a follow-up longer than three months were included for survival analysis. In addition, patients with prior treatment such as neoadjuvant chemotherapy and surgery were excluded.

### Differentially-expressed, immunity-related gene analysis

To identify IRGs involved in breast cancer progression, we first screened differentially expressed genes (DEGs) between tumoral and normal tissue. FPKM data were transformed in the form of log_2_ (FPKM+0.001), |log2 fold change| > 1 and false discovery rates (FDR) < 0.05 were set as the cutoff values. A list of 2498 immunity-related genes was obtained from the Immunology Database and Analysis Portal (ImmPort) [54]. Then, the immunity-related gene list and DEGs were intersected to extract differentially-expressed IRGs. We performed hierarchical clustering analysis to visualize the expression patterns of the differentially-expressed IRGs between normal and breast cancer tissues.

### Functional enrichment analysis

To further annotate the biological function of IRGs of interest, we carried out enrichment analysis of Kyoto Encyclopedia of Genes and Genomes (KEGG) pathway [55] and Gene Ontology (GO) including biological process (BP), molecular function (MF) and cellular component (CC) genes by using the well-established DAVID Bioinformatics Resources [56]. *P* values were adjusted by the False-Discovery Rate (FDR) method for multiple comparisons and pathways with FDR < 0.05 were considered enriched.

### Construction of the IRGs-based prognostic signature

Univariate Cox regression analysis was performed using the “survival package” of R to investigate the prognostic value of differentially-expressed IRGs in breast cancer patients. Ones with P < 0.05 were considered survival-associated IRGs, which were selected for multivariate Cox regression analyses to obtain independent prognostic IRGs and construct an immunity-based prognostic model. The risk score of each patient was the sum of products of the expression value and the regression coefficient of each independent prognostic IRG, $\text{risk}\;\text{score}=\sum_{i=1}^{n}\beta_{i}Exp_{i}$. Patients were then divided into high- and low-risk groups according to the median risk score. Survival analysis was performed by the Kaplan-Meier method and the log-rank test was utilized to compare the statistical significance of the difference between high- and low-risk groups.

Additionally, univariate and multivariate analyses were conducted to evaluate the effects of risk score and other critical clinical features on patient overall survival.

### Regulatory network analysis between transcription factors and survival-associated IRGs

The Cistrome Cancer web resource enables us to investigate regulatory links between TFs and transcriptomes in cancer by integrating public cancer genomics data with chromatin profiling data [57]. We downloaded a total of 318 TFs from Cistrome Cancer, according to which we screened differentially-expressed TFs from the DEGs. Correlation tests were conducted between differentially-expressed TFs and survival-associated IRGs, and the regulatory network between them was visualized by using Cytoscape.

### Association analysis between immunity-related signature and immune infiltrates

The correlation of the risk score of each patient with immune cell infiltration was analyzed by virtue of Tumor Immune Estimation Resource (TIMER), which is an online resource for systematical analysis in diverse cancers of immune infiltrating cells, including B cells, CD4+ T cells, CD8+ T cells, neutrophils, macrophages and dendritic cells [58]. We used TIMER-estimated abundances of tumor-infiltrating immune cells to analyze their associations with IRGs-based prognostic signature in breast cancer.

### Gene set enrichment analysis

To investigate immune response processes affected by the hub IRGs in the prognostic signature, we performed gene set enrichment analysis (GSEA) between high- and low-risk groups. Immunity-related gene sets in GO terms were downloaded from the Molecular Signatures Database (MSigDB) for GSEA analysis. A gene set was considered enriched for *P* < 0.05 and FDR < 0.25.

### Statistical analysis

All analyses were performed using R software (version 3.4.4), except for GSEA analysis. Unless otherwise indicated, differences were considered statistically significant when *P* < 0.05.

## Supplementary Material

Supplementary Table 1 and 3

Supplementary Table 2
