# Supplementary material for: Construction and validation of an immunity-related prognostic signature for breast cancer
Source: Aging (Albany NY). 2020 Nov 7;12(21):21597–612. doi: 10.18632/aging.103952 (PMC7695418; doi:10.18632/aging.103952)
Supplement: Supplementary Table 1 and 3 [file aging-12-103952-s001..pdf]

## SUPPLEMENTARY TABLES

**Supplementary Table 1. Differentially expressed immunity-related genes identified between breast cancer and normal tissues.**

| Regulation | Gene symbol |
|---|---|
| Up-regulated (69) | *CST4, CGA, VGF, CCL11, SCT, FABP6, S100A7A, TUBB3, CCL7, CCR8, IGHE, CAMP, PRLH, ORM1, TFR2, ULBP1, EPO, TNFRSF9, UMODL1, S100P, PROC, ARTN, OPRD1, IL21R, AMH, CXCL11, BIRC5, BMP8A, IL20, IL9R, CD1A, ORM2, ESM1, INHBA, PCSK1, PYY, IL27, TNFRSF18, BMPR1B, LTA, TRH, CCL20, ICOS, TMPRSS6, KCNH2, TNFSF4, EDN2, IL11, CCR4, ADM2, PDCD1, LCN12, ANGPTL6, WFIKKN1, CD19, OLR1, CXCL10, SCG2, SLC11A1, MMP12, TRAV26-1, OASL, PIK3R2, FGFR3, THPO, ULBP2, RBP2, TRAV39, INHA* |
| Down-regulated (110) | *ANGPTL7, ACVR1C, DEFB132, LEP, TSLP, LALBA, PENK, GDF10, RNASE7, EDN3, GFAP, RXRG, CCL14, SSTR1, PAK3, ADCYAP1R1, GPR17, CCL24, NTF4, NRG2, CXCL2, CMA1, ADIPOQ, AVPR2, TACR1, KL, PLXNA4, IL17B, BMP5, ANGPTL1, RBP4, OXTR, PROK1, NRG1, ANGPT1, DES, CXCL3, FAM3D, ALB, IGF1, ADRB2, PTGFR, FGF2, LEPR, SEMA3D, GREM2, BMP2, PTGS2, SAA2, S100B, CNTFR, IL6, TAC1, NTS, CD209, NPR1, CCL23, ADRB1, PTH1R, IL22RA2, PPARG, NR3C2, IL33, FABP4, PI15, PTX3, ROBO3, SEMA3A, SEMA6D, PI3, NTF3, GHR, FABP7, BMP6, TNFRSF10D, OGN, CTSG, LGR6, EGFR, LIFR, NR4A3, CCL13, SCGB3A1, SPINK5, CXCL5, MASP2, EDNRB, CCL28, IL17D, TGFBR3, SHC3, NPR3, NGFR, FGF10, FGF7, CXCL6, MIA, PTH2R, BACH2, NR5A2, FGF16, SEMA3G, FGF1, GNAI1, MARCO, CCL21, DEFB1, TNFRSF8, SAA1, S100A12* |

**Supplementary Table 3. Correlation of risk score with tumor stage in breast cancer.**

| Tumor stage | Number of patients | Risk score | *P* |
|---|---|---|---|
| i-iia | 497 | 1.205 ± 1.011 | 0.044 |
| iib-iv | 466 | 1.338 ± 1.054 | |
