# Supplementary material for: Construction and validation of an immunity-related prognostic signature for breast cancer
Source: Aging (Albany NY). 2020 Nov 7;12(21):21597–612. doi: 10.18632/aging.103952 (PMC7695418; doi:10.18632/aging.103952)
Supplement: Supplementary Table 2 [file aging-12-103952-s002..pdf]

Supplementary Table 2. Functional enrichment analysis for differentially expressed immunity-related genes in breast cancer.

Category	Term ID	Term name	Count	FDR	Gene symbol
KEGG	hsa04060	Cytokine-cytokine receptor interaction	42	3.18E-28	*IL9R, CXCL5, CXCL3, LEPR, IL21R, CXCL2, TNFRSF8, CNTFR, CXCL6, CXCL11, CCL28, CCL7, IL11, CXCL10, CCL24, IL17B, CCL23, CCL20, CCL21, TNFRSF18, LTA, GHR, EPO, THPO, IL6, BMP2, TNFSF4, LIFR, IL20, CCL11, LEP, TSLP, TNFRSF9, INHBA, CCR8, AMH, CCL13, CCL14, TNFRSF10D, CCR4, NGFR, BMPR1B*
KEGG	hsa04062	Chemokine signaling pathway	20	1.96E-07	*CXCL5, GNAI1, CXCL3, CXCL2, CXCL6, CXCL11, CCL28, CCL7, CXCL10, CCL24, CCL11, CCR8, CCL13, CCL23, CCL14, CCL20, CCR4, CCL21, SHC3, PIK3R2*
KEGG	hsa04630	Jak-STAT signaling pathway	15	0.000126	*IL6, IL9R, LEPR, IL21R, LIFR, CNTFR, IL20, IL11, LEP, TSLP, GHR, IL22RA2, PIK3R2, THPO, EPO*
KEGG	hsa04080	Neuroactive ligand-receptor interaction	17	0.015305	*AVPR2, CGA, PTH2R, LEPR, TACR1, ADCYAP1R1, PTH1R, OXTR, PTGFR, LEP, EDNRB, ADRB2, ADRB1, SSTR1, CTSG, GHR, OPRD1*
KEGG	hsa04923	Regulation of lipolysis in adipocytes	8	0.0431	*CGA, ADRB2, ADRB1, PTGS2, GNAI1, NPR1, FABP4, PIK3R2*
GO BP	GO:0006954	inflammatory response	37	2.37E-21	*CXCL5, PTGS2, TACR1, CXCL3, CXCL2, TAC1, TNFRSF8, CXCL6, CXCL11, CCL7, CXCL10, CCL24, SLC11A1, IL17D, IL17B, CCL23, CCL20, CCL21, TNFRSF18, PTX3, SCG2, IL6, BMP2, OLR1, IL27, PTGFR, S100A12, CCL11, TNFRSF9, ORM1, CCL13, CCL14, TNFRSF10D, CCR4, NGFR, BMPR1B, BMP6*
GO BP	GO:0007267	cell-cell signaling	31	4.44E-20	*LALBA, CGA, EDN3, FGFR3, CXCL5, FGF16, TAC1, CXCL6, TRH, CXCL11, CCL7, IL11, CXCL10, CCL24, PCSK1, IL17B, CCL23, CCL20, CCL21, LTA, BMP2, NTF4, NTF3, INHA, INHBA, AMH, ADRB2, CCL13, ADRB1, SSTR1,*

					PYY
GO BP	GO:0006955	immune response	36	9.48E-19	*CXCL5, CXCL3, CXCL2, TNFRSF8, CXCL6, CXCL11, CCL28, CXCL10, CCL24, SLC11A1, IL17B, CCL23, CCL20, CCL21, ICOS, TNFRSF18, DEFB1, LTA, IL6, TNFSF4, CD1A, IL20, CCL11, CCR8, TNFRSF9, CCL13, CCL14, TNFRSF10D, CCR4, IGHE, TGFBR3, CMA1, NGFR, CTSG, BMP6, OPRD1*
GO BP	GO:0070098	chemokine-mediated signaling pathway	17	2.78E-14	*CXCL5, CXCL3, CXCL2, CXCL6, CXCL11, CCL7, CXCL10, CCL24, CCL11, CCR8, CCL13, CCL23, CCL14, CCL20, CCL21, CCR4, GPR17*
GO BP	GO:0032496	response to lipopolysaccharide	20	1.11E-11	*CXCL5, PTGS2, CXCL3, CXCL2, TAC1, TNFRSF8, FGF10, CXCL6, CXCL11, PTGFR, CXCL10, SLC11A1, TNFRSF9, PENK, TNFRSF10D, TNFRSF18, NGFR, CTSG, LTA, EPO*
GO BP	GO:0008284	positive regulation of cell proliferation	30	1.11E-11	*CGA, EDN3, AVPR2, FGF7, FGFR3, CXCL5, EDN2, PTH1R, CNTFR, ESM1, IL11, CXCL10, EDNRB, NRG1, FGF1, FGF2, EPO, EGFR, IL6, TNFSF4, NTF3, CAMP, LIFR, IGF1, BIRC5, PTGFR, LEP, CCL14, S100B, PROK1*
GO BP	GO:0006935	chemotaxis	16	3.65E-09	*CXCL5, CXCL2, CXCL6, CXCL11, CCL28, CCL7, CXCL10, CCL24, CCL11, CCR8, CCL13, CCL23, CCL20, CCR4, DEFB1, FGF2*
GO BP	GO:0070374	positive regulation of ERK1 and ERK2 cascade	18	5.75E-09	*EGFR, IL6, BMP2, FGFR3, FGF10, CCL7, CCL24, CCL11, CCL13, CCL23, CCL14, CCL20, CCL21, ANGPT1, FGF1, FGF2, EPO, THPO*
GO BP	GO:0007166	cell surface receptor signaling pathway	21	1.36E-08	*EGFR, EDN3, PTH2R, LEPR, EDN2, TACR1, ADCYAP1R1, PTH1R, LIFR, OXTR, NPR1, INHA, CXCL10, MARCO, INHBA, IL17D, EDNRB, ADRB2, CD19, IL17B, SSTR1*
GO BP	GO:0007165	signal transduction	40	6.6E-08	*LALBA, CGA, EDN3, IL9R, FGF7, CXCL5, PPARG, FGF16, NR3C2, TNFRSF8, CNTFR, CXCL6, TRH, CXCL11, CCL7, PDCD1, CXCL10, CCL24, CCL23, CCL20, TNFRSF18, SHC3, FGF1, FGF2, NRG2, LTA, EPO, PIK3R2, EGFR, TNFSF4, NTF3, ARTN, IGF1, INHA, CCL11, LEP, CCL13, NTS, PENK, TNFRSF10D*

GO BP	GO:0048015	phosphatidylinositol-mediated signaling	14	1.17E-07	*EGFR, FGF7, FGFR3, KL, FGF16, FGF10, IGF1, NPR3, CD19, FGF1, NRG1, NRG2, FGF2, PIK3R2*
GO BP	GO:0030593	neutrophil chemotaxis	12	1.18E-07	*CCL24, CCL11, EDN3, CCL13, CCL23, CCL14, CCL20, SAA1, EDN2, CXCL3, CCL7, S100A12*
GO BP	GO:0002548	monocyte chemotaxis	10	6.22E-07	*CCL24, CCL11, CCL13, IL6, CCL23, CCL14, CCL20, CCL21, CCL7, S100A12*
GO BP	GO:0014066	regulation of phosphatidylinositol 3-kinase signaling	12	7.68E-07	*EGFR, CD19, FGFR3, FGF7, KL, FGF16, FGF10, FGF1, NRG1, FGF2, NRG2, PIK3R2*
GO BP	GO:0046854	phosphatidylinositol phosphorylation	12	5.91E-06	*EGFR, CD19, FGFR3, FGF7, KL, FGF16, FGF10, FGF1, NRG1, FGF2, NRG2, PIK3R2*
GO BP	GO:0050729	positive regulation of inflammatory response	11	6.52E-06	*CCL24, CCL11, EGFR, CCL13, CCL23, CCL14, TNFSF4, FABP4, IL33, CCL7, S100A12*
GO BP	GO:0048247	lymphocyte chemotaxis	8	1.87E-05	*CCL24, CCL11, CCL13, CCL23, CCL14, CCL20, SAA1, CCL21*
GO BP	GO:0002690	positive regulation of leukocyte chemotaxis	7	3.18E-05	*EDN3, IL6, CXCL5, EDN2, CXCL6, CXCL11, CXCL10*
GO BP	GO:0060326	cell chemotaxis	10	3.64E-05	*CCL13, CCL14, SAA2, CXCL5, CCL20, CCL21, CXCL2, CXCL6, CCL28, CXCL10*
GO BP	GO:0007186	G-protein coupled receptor signaling pathway	30	7.66E-05	*CXCL5, GNAI1, ADCYAP1R1, CXCL3, PTH1R, CXCL2, PPARG, OXTR, CXCL6, CXCL11, LGR6, CCL7, CXCL10, CCL24, CCL23, CCL20, CCL21, DEFB1, PRLH, PTH2R, NPR1, PTGFR, CCL11, CCR8, CCL13, CCL14, CCR4, GPR17, PYY, OPRD1*
GO BP	GO:0071347	cellular response to interleukin-1	10	8.04E-05	*CCL24, CCL11, CCL13, IL6, CCL23, CCL14, CCL20, CCL21, CAMP, CCL7*
GO BP	GO:0050918	positive chemotaxis	8	0.0001	*FGF7, NTF3, SAA2, SAA1, FGF10, ANGPT1, FGF2, SCG2*

GO BP	GO:0000187	activation of MAPK activity	11	0.000272	*BMP2, NTF3, PAK3, SAA1, PROK1, IGF1, FGF10, FGF1, NRG1, FGF2, GHR*
GO BP	GO:0050731	positive regulation of peptidyl-tyrosine phosphorylation	10	0.000287	*IL6, FGF7, NTF3, IGF1, FGF10, ANGPT1, NRG1, ADIPOQ, GHR, IL11*
GO BP	GO:0050679	positive regulation of epithelial cell proliferation	9	0.000306	*EGFR, IL6, FGF7, IGF1, FGF10, NR4A3, FGF1, BMP5, BMP6*
GO BP	GO:0071356	cellular response to tumor necrosis factor	11	0.000354	*CCL24, CCL11, CCL13, IL6, CCL23, CCL14, CCL20, CCL21, CAMP, FABP4, CCL7*
GO BP	GO:0043547	positive regulation of GTPase activity	22	0.000695	*EGFR, FGFR3, FGF7, KL, FGF16, FGF10, ARTN, CCL7, CCL24, CCL11, CCL13, CCL23, CCL14, ADRB1, CCL20, ANGPT1, FGF1, NRG1, SHC3, NRG2, FGF2, PIK3R2*
GO BP	GO:0030335	positive regulation of cell migration	13	0.000814	*CCL24, CCL11, EGFR, CGA, BMP2, NTF3, SEMA6D, SEMA3G, SEMA3D, IGF1, FGF1, LGR6, CCL7*
GO BP	GO:0000165	MAPK cascade	15	0.001016	*EGFR, FGFR3, FGF7, KL, FGF16, ARTN, FGF10, PAK3, ANGPT1, FGF1, SHC3, NRG1, NRG2, FGF2, SCG2*
GO BP	GO:0036092	phosphatidylinositol-3-phosphate biosynthetic process	8	0.001132	*FGFR3, FGF7, KL, FGF16, FGF10, FGF1, FGF2, PIK3R2*
GO BP	GO:0048245	eosinophil chemotaxis	5	0.002254	*CCL24, CCL11, CCL13, CCL7, SCG2*
GO BP	GO:0001501	skeletal system development	11	0.00272	*WFIKKN1, BMP2, FGFR3, PTH1R, IGF1, GDF10, INHA, NPR3, BMPR1B, BMP5, BMP6*
GO BP	GO:0009409	response to cold	7	0.002859	*IL6, ADRB2, ADRB1, PPARG, TRH, VGF, CXCL10*
GO BP	GO:0043410	positive regulation of MAPK cascade	9	0.003198	*LEP, BMP2, IL6, ADRB2, FGFR3, IGF1, FGF10, NGFR, IL11*

GO BP	GO:0071346	cellular response to interferon-gamma	8	0.003249	*CCL24, CCL11, CCL13, CCL23, CCL14, CCL20, CCL21, CCL7*
GO BP	GO:0006953	acute-phase response	7	0.004667	*ORM1, IL6, SAA2, SAA1, TFR2, ORM2, EPO*
GO BP	GO:0010862	positive regulation of pathway-restricted SMAD protein phosphorylation	7	0.016248	*INHBA, BMP2, GDF10, INHA, BMP5, BMP8A, BMP6*
GO BP	GO:0007204	positive regulation of cytosolic calcium ion concentration	10	0.018019	*EDNRB, CCR8, SAA1, CCR4, EDN2, PTH1R, TAC1, OXTR, PTGFR, CCL28*
GO BP	GO:0030509	BMP signaling pathway	8	0.022775	*BMP2, TGFBR3, GDF10, BMPR1B, GREM2, BMP5, BMP8A, BMP6*
GO BP	GO:0042742	defense response to bacterium	10	0.034033	*LALBA, SLC11A1, DEFB132, CCL20, CAMP, IGHE, CXCL6, VGF, DEFB1, S100A12*
GO BP	GO:0001525	angiogenesis	12	0.035755	*LEP, ANGPTL6, PTGS2, PROK1, LEPR, FGF10, ANGPT1, ADM2, ESM1, FGF1, TMPRSS6, SCG2*
GO BP	GO:0050829	defense response to Gram-negative bacterium	7	0.036147	*LALBA, SLC11A1, IL6, RNASE7, CAMP, TAC1, DEFB1*
GO BP	GO:0042127	regulation of cell proliferation	11	0.039403	*TNFRSF10D, CXCL3, CXCL2, TNFRSF18, SCGB3A1, NGFR, CXCL6, INHA, CXCL11, NR5A2, CXCL10*
GO BP	GO:0051897	positive regulation of protein kinase B signaling	8	0.044015	*LEP, EGFR, IL6, CCL21, ANGPT1, NRG1, FGF2, THPO*
GO CC	GO:0005576	extracellular region	94	3.01E-48	*CGA, EDN3, FGF7, IL9R, FAM3D, MASP2, LEPR, EDN2, FGF16, FGF10, CXCL11, SPINK5, IL11, CXCL10, SCT, OGN, SAA1, RNASE7, ANGPT1,*

					SEMA3A, FGF1, NRG1, NRG2, FGF2, PRLH, GHR, IL27, CAMP, ARTN, INHA, MMP12, IL20, PROC, INHBA, AMH, PROK1, IGHE, NGFR, CTSG, RBP4, WFIKKN1, FGFR3, CXCL5, CXCL3, CXCL2, TAC1, CXCL6, IL33, ESM1, TRH, CCL28, GREM2, CCL7, ANGPTL7, IL17D, CCL23, IL17B, CCL20, CCL21, ALB, ICOS, TNFRSF18, PTX3, DEFB1, EPO, THPO, BMP2, IL6, LCN12, NTF4, DEFB132, NTF3, KL, IGF1, PTGFR, ADIPOQ, S100A12, CCL11, LEP, ORM1, CCL13, CCL14, PENK, NTS, S100B, TGFBR3, CMA1, ADM2, PYY, BMP5, ORM2, IL22RA2, BMP8A, BMP6
GO CC	GO:0005615	extracellular space	84	7.17E-44	*EDN3, IL9R, EDN2, FGF16, FGF10, VGF, CXCL11, IL11, CXCL10, SCT, OGN, SAA2, SEMA3G, SAA1, SEMA3D, ANGPT1, SEMA3A, FGF1, NRG1, NRG2, FGF2, LTA, GHR, EGFR, IL27, CAMP, ARTN, IL20, PROC, TNFRSF9, AMH, UMODL1, CST4, ULBP2, SCGB3A1, CTSG, LALBA, MIA, RBP4, CXCL5, CXCL3, CXCL2, TAC1, CXCL6, IL33, CCL28, GREM2, CCL7, CCL24, IL17D, PCSK1, CCL23, IL17B, CCL20, CCL21, ALB, ANGPTL1, PTX3, DEFB1, SCG2, EPO, THPO, BMP2, IL6, TNFSF4, KL, IGF1, ADIPOQ, TMPRSS6, CCL11, LEP, ORM1, TSLP, CCL13, CCL14, S100B, TGFBR3, GDF10, PYY, BMP5, ORM2, IL22RA2, BMP8A, BMP6*
GO CC	GO:0005887	integral component of plasma membrane	41	5.7E-07	*AVPR2, IL9R, FGFR3, PLXNA4, TFR2, ADCYAP1R1, TACR1, PTH1R, OXTR, TNFRSF8, LGR6, MARCO, SLC11A1, EDNRB, ICOS, TNFRSF18, NRG1, GHR, TNFSF4, OLR1, PTH2R, KL, LIFR, NPR1, CD1A, NPR3, PTGFR, CCR8, TNFRSF9, ADRB2, ADRB1, CD19, SEMA6D, TNFRSF10D, SSTR1, CCR4, TGFBR3, GPR17, NGFR, BMPR1B, OPRD1*
GO CC	GO:0043235	receptor complex	12	5.03E-05	*EGFR, ADRB2, OLR1, LEPR, ADCYAP1R1, PTH1R, NR3C2, LIFR, NPR1, TGFBR3, BMPR1B, GHR*
GO CC	GO:0009897	external side of plasma membrane	12	0.008722	*TNFRSF9, IL6, CD19, UMODL1, CCR4, ICOS, TFR2, IGHE, TGFBR3, NRG1, PDCD1, CXCL10*

GO MF	GO:0008083	growth factor activity	27	4.83E-21	*MIA, IL6, BMP2, NTF4, FGF7, NTF3, FGF16, ARTN, FGF10, IGF1, INHA, VGF, IL11, LEP, OGN, AMH, INHBA, PROK1, GDF10, FGF1, NRG1, NRG2, FGF2, BMP5, BMP6, BMP8A, THPO*
GO MF	GO:0005125	cytokine activity	25	1.64E-17	*IL6, BMP2, TNFSF4, FAM3D, IL27, IL33, INHA, GREM2, ADIPOQ, IL11, IL20, TSLP, IL17D, INHBA, IL17B, GDF10, SCGB3A1, NRG1, FGF2, LTA, BMP5, SCG2, BMP6, BMP8A, THPO*
GO MF	GO:0008009	chemokine activity	15	3.85E-14	*CXCL5, CXCL3, CXCL2, CXCL6, CXCL11, CCL28, CCL7, CXCL10, CCL24, CCL11, CCL13, CCL23, CCL14, CCL20, CCL21*
GO MF	GO:0005179	hormone activity	17	1.49E-12	*EDN3, CGA, KL, EDN2, IGF1, INHA, VGF, ADIPOQ, LEP, AMH, SCT, INHBA, ADM2, PYY, PRLH, THPO, EPO*
GO MF	GO:0046934	phosphatidylinositol-4,5-bisphosphate 3-kinase activity	12	3.2E-08	*EGFR, CD19, FGFR3, FGF7, KL, FGF16, FGF10, FGF1, NRG1, FGF2, NRG2, PIK3R2*
GO MF	GO:0005088	Ras guanyl-nucleotide exchange factor activity	14	1.73E-07	*EGFR, FGFR3, FGF7, KL, FGF16, ARTN, FGF10, ADRB1, ANGPT1, FGF1, SHC3, NRG1, NRG2, FGF2*
GO MF	GO:0005160	transforming growth factor beta receptor binding	9	1.01E-05	*AMH, INHBA, BMP2, TGFBR3, GDF10, INHA, BMP5, BMP8A, BMP6*
GO MF	GO:0008201	heparin binding	14	1.05E-05	*FGF7, FGF10, CXCL6, CXCL11, GREM2, CCL7, CXCL10, OGN, CCL23, SAA1, TGFBR3, FGF1, FGF2, CTSG*
GO MF	GO:0016303	1-phosphatidylinositol-3-kinase activity	8	0.000284	*FGFR3, FGF7, KL, FGF16, FGF10, FGF1, FGF2, PIK3R2*
GO MF	GO:0042056	chemoattractant activity	7	0.000307	*FGF7, NTF3, SAA2, SAA1, FGF10, FGF2, SCG2*
GO MF	GO:0017046	peptide hormone	7	0.000307	*EDNRB, INHBA, PTH1R, NPR1, OXTR, NPR3, GHR*

		binding			
GO MF	GO:0005102	receptor binding	17	0.000715	*EDN3, BMP2, TNFSF4, NTF3, IL27, ARTN, TAC1, CNTFR, INHA, ADIPOQ, CXCL10, AMH, CCL13, ANGPTL1, NRG1, NRG2, LTA*
GO MF	GO:0005104	fibroblast growth factor receptor binding	6	0.002917	*FGF7, KL, FGF16, FGF10, FGF1, FGF2*
GO MF	GO:0048020	CCR chemokine receptor binding	6	0.003698	*CCL24, CCL11, CCL13, CCL14, CCL20, CCL21*
GO MF	GO:0005184	neuropeptide hormone activity	6	0.014796	*NTS, PENK, PYY, TRH, VGF, PRLH*
